# American Journal of Urology

**Published:** 1905-11

**Authors:** 


### American Journal of Urology.

DR. HENRY G. SPOONER, of New York, who has been editor of the *American Journal of Urology* since its first issue, has retired from its editorial management. Dr. Spooner in spite of the many difficulties and embarrassments attending the inception of a new publication, made a brilliant success of the *Journal.* Dr. Spooner's effort was always to make it a practical publication, and with the assistance of Dr. Frederick Bierhoff, who was in charge of the abstract department, most admirably succeeded. Dr. Spooner secured the best literature in the genitourinary field in this country and Europe, and it is due to his efforts that the *Journal* has reached the high literary and professional standard that it now occupies.

Dr. Charles Greene Cumston, of Boston, has been named by the publication committee of the American Urological Association as Dr. Spooner's successor. The *Journal,* therefore, will hereafter be edited from Boston.

---

RECIPROCITY in state medical licensure between New York and New Jersey has been settled definitly by a protocol signed by the representatives of each commonwealth, to take effect January 1, 1906. The secretary of the New Jersey board announces that New Jersey will endorse the medical license issued by any State, after examination, whose educational examination and licensing requirements are substantially equal to, or higher than, those of New Jersey, irrespective of reciprocity, provided the applicant complies with the conditions of indorsement.

The language of the New York statute bearing on this point is as follows:

Applicants examined and licensed by other state examining boards registered by the Regents as maintaining standards not lower than those provided by this article, may without further examination, on payment of $10 to the Regents and on submitting such evidence as they may require, receive from them an indorsement of their licenses or diplomas conferring all rights and privileges of a Regents license issued after examination.

Heretofore New Jersey has charged a fee of $50 to indorse the New York license.

---

THE university extension plans progress with reasonable speed. Under the leadership of Vice-Chancellor Norton, who is supported by many of our foremost citizens, a substantial advance has been made already, and forces are at work on all sides to enlist other aid that may afford a further financial support to the scheme. The JOURNAL hopes for the success of the proposition to establish the University of Buffalo in fast as well a in name and offers the unrestricted use of its columns to thhat end.

---

THE JOURNAL has been embarrassed in printing the issues of October and November by reason of a "strike" of compositors in the printing offices in Buffalo. We feel all the more annoyed at this delay because of the rule of promptitude we established at the beginning of our editorial management and which we have maintained until the present time. If our readers will indulge us a little further we think that before January we shall be able to resume our former punctuality.

---

Doctor—Well, John, how are you to-day?

John—Verra bad, verra bad. I wish Providence 'ud 'ave mussy on me an' take me!

Wife—'Ow can you expect it to if you won't take the doctor's physic?—*Punch*.

---

Thoroughly Sterilized—Aunt Beth—They say his money is tainted!

Edith—Nonsense, aunty; I heard him say he had just cleaned up another million!—*Puck*.

